# Preliminary study of thermal density distribution and entropy analysis during cycling exercise stress test using infrared thermography

**DOI:** 10.1038/s41598-022-18233-5

**Published:** 2022-08-18

**Authors:** S. Bogomilsky, O. Hoffer, G. Shalmon, M. Scheinowitz

**Affiliations:** 1 Sylvan Adams Sports Institute, School of Public Health, Sackler Faculty of Medicine, Tel Aviv University, 6997801 Tel Aviv, Israel; 2 School of Electrical Engineering, Afeka Tel Aviv Academic College of Engineering, 6910717 Tel Aviv, Israel; 3 Department of Biomedical Engineering, Faculty of Engineering, Tel Aviv University, 6997801 Tel Aviv, Israel

**Keywords:** Biomedical engineering, Physiology

## Abstract

Considerable differences related to the results of temperature changes acquired during exercise exist, and in many cases, these lead to poor correlation with physiological variables. In this preliminary study we investigated the temperature changes and the temperature distribution (entropy) of the torso during a graded cycling exercise stress test using thermal imaging and studied the correlation between the increase in pulmonary ventilation (VE) and the changes in the surface temperature of the anterior torso during exercise. Thermal images of the anterior torso were captured every 30 s during the exercise, while the resistance was gradually increased every minute until exhaustion. The thermal images were processed to obtain a mean temperature in the regions of interest (ROI) (chest, forehead, and abdomen). We also developed an algorithm to calculate the distribution of temperature and texture (entropy) within each ROI. No changes were found in absolute temperatures. However, the entropy of the chest surface area increased significantly throughout the exercise test, compared with baseline temperature at rest. This increase in entropy was significantly correlated with exercise duration and intensity (p < 0.001). Furthermore, a high correlation between the increase in VE and chest entropy during exercise was detected (r = 0.9515). No correlations were found between the increase in entropy and the abdomen or the forehead compared with the VE. The non-invasive IR thermal imaging during graded exercise, combined with advanced image processing, successfully correlates surface thermography with exercise duration and pulmonary ventilation.

## Introduction

Thermal imaging is based on the detection of electromagnetic radiation in the infrared range to measure changes in body surface radiation temperature^1,2^. This method is non-invasive, radiation-free, cheap, and easy to use. The use of thermal imaging and image processing algorithms has recently increased in the medical field including, diagnosis, exercise physiology, and sports medicine^1–6^.

There are considerable differences in the literature regarding the results of temperature changes during exercise, with only a few studies hinting towards correlation with physiological parameters^1^. Akimov et al.^7,8^ examined the forehead temperature changes during exercise and observed a correlation of the 4 mmol lactate threshold (the lactic acid level where production and clearance are disrupted, resulting in an accumulation of lactic acid in the blood) with the beginning of an increase in the forehead skin temperature. Duc et al.^9^ observed a relationship between heart rate and oxygen uptake during exercise. They also observed a correlation between skin temperature variations of the thigh and gluteus maximus muscles.

Additionally, previous studies described temperature distribution changes in the anterior torso during incremental effort as "tree-shaped" or "hyper-thermal" surface radiation patterns using enhanced thermal imaging analysis^10–12^. Warmer areas appeared brighter in thermal photography, creating a characteristic image reminiscent of a tree and its branches. To the best of our knowledge, these changes were only observed in the images and not in the numerical or statistical calculations.

In this preliminary study, we examined the torso's temperature changes and the temperature distribution (entropy) during a graded cycling exercise stress test using thermal imaging. The study also investigated a correlation between the increase in pulmonary ventilation (VE) during exercise and the corresponding changes in the surface temperature of the anterior torso. We hypothesized that the activation of the chest muscles during incremental exercise and the associated increase in tidal volume, breathing frequency and, overall cost of breathing would be accompanied by comparable changes in the surface temperature.

## Methods

The study was approved by the Ethics Committee of Tel Aviv University (approval number: 0003310-1). All study participants provided informed consent. All methods were performed in accordance with the relevant guidelines and regulations. research was conducted at the Sylvan Adams Sports Institute at Tel Aviv University. The sample size was calculated with 0.05 alpha and 80% power. Eighteen healthy young men with a mean age of 24.50 ± 1.88 years, height 179.90 ± 8.50 cm and body mass 75.80 ± 9.49 kg participated in the study. The study was performed only on men because base-chest images needed to be acquired. The participants performed an incremental exercise stress test in the sitting position on an SRM stationary ergometer (Schoberer Rad-Messtechnik, SRM GmbH, Germany). Each time, the bicycle was adjusted to each participant's height before the test was initiated. Following 5 min of warm-up, the experimental protocol started with a resistance of 80 W. The resistance was increased by 20 W each minute until exhaustion. After the test, the participants remained sitting on the bike for 5 min of recovery. The test included gas exchange measurements using a COSMED Quark CPET cardiometabolic system (Italy).

Thermal images of the chest were captured at rest, every 30 s during the exercise stress test, and every 60 s during the 5-min recovery period.

The CPET data were processed. The aerobic threshold (AT) and the respiratory compensation (RC) points for each participant were determined. Subsequently, the thermal images were analyzed and compared with the changes in VE over time, and with the AT and the RC points.

We have used Binder’s et al. method to detect the aerobic (AT) and the anaerobic (RC) thresholds^13^. Pulmonary ventilation increases during exercise stress test while showing two break points. The first one (VT1—ventilatory threshold 1) is referred to the ‘aerobic threshold’ and the second one (VT-2), to the ‘anaerobic threshold’. VT2 is also referred as Ventilatory Compensation (RC) point^13^.

### Thermal imaging

A FLIR ONE thermal camera device (Teledyne FLIR LLC. Wilsonville, OR, USA) was used to acquire the thermal images. FLIR ONE connects directly to smartphones and utilizes the following functions: a frame rate frequency of 8.7 Hz, an object temperature range of − 20 °C to 120 °C, and thermal sensitivity of 100 mK. The FLIR ONE Thermal resolution is 160 × 120. The wavelength sensitivity over which the camera interpolates the temperature was 8–14 µm, and the emissivity value considered appropriate for accurate human temperature readings was 0.98. The room temperature maintained at 21.1 ± 0.6° and the humidity was 62.7 ± 2.1%.

The optimal imaging procedure included images of the anterior torso of the participant (chest, abdomen, and forehead). The participants were asked to remove their top clothing and sit upright. The angle of the image was maintained at a constant value with the camera set parallel to the participant's body (anterior view). Images were captured at a distance which was maintained constant for all tests at approximately 80 cm. Images (20 to 40) were acquired for each participant, which were then processed for downstream analysis. A total of 423 images were processed.

### Thermal image processing

**Figure 1:**
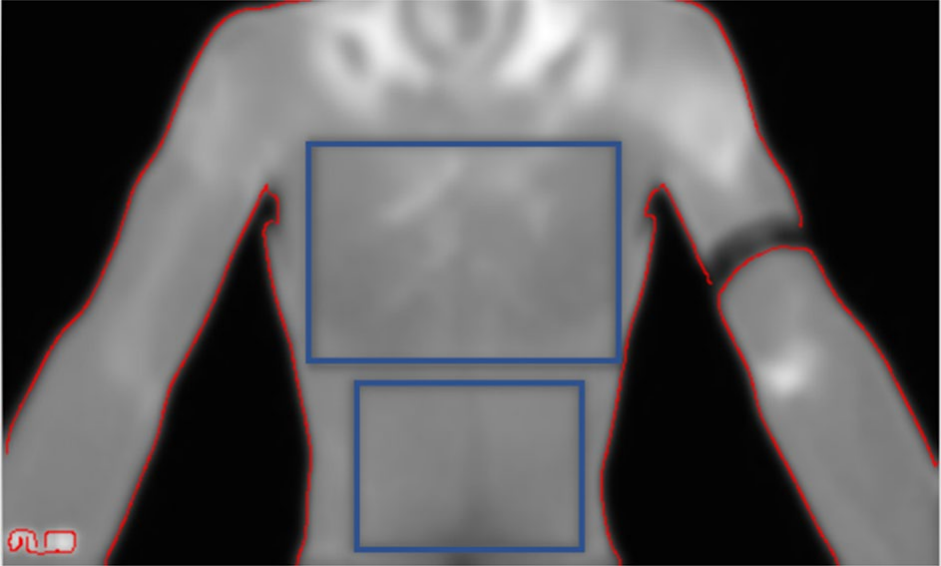
Manually selected ROIs for the chest and abdomen.

We used MATLAB software (Mathworks Inc. Natick, MA, USA) for image processing and entropy analysis. The software first reads a matrix with temperature values in the entire region. This temperature map is then displayed, and a region of interest (ROI) is manually selected (in our research forehead, chest, and abdomen). Then, the algorithm computes the mean temperature in the ROI and entropy (Fig. 1).

Additional textural parameters were computed using MATLAB commands. The set of temperatures was transformed into non-negative integers with a difference of 0.1 °C between adjacent values for computing these parameters. The temperatures were first rounded to a precision of 0.1 °C. Finally, the minimum temperature in the ROI was subtracted from all the values.

### Temperature distribution and entropy

Entropy is a statistical measure of randomness used to characterize the texture of an input image and can be expressed as:

1 $$H\left(x\right)=-{\sum }_{k=1}^{n}p\left({\mathrm{x}}_{\mathrm{k}}\right){\mathrm{log}}_{2}p({\mathrm{x}}_{\mathrm{k}})$$

The entropy of each ROI was computed using Eq. (1).

The algorithm developed for this study computed and stored the temperature distribution and entropy for each selected ROI for each subject.

### Statistical analysis

Variables were expressed as a median and at 95% confidence intervals. The results of each participant (CPET results and those of the thermal images) were normalized according to the exercise duration. The end of the exercise was set as 100%, and the values that represented the percentages of the maximum effort time were calculated. Then, the mean of all participants was calculated according to the normalized values (the specific statistical tests are detailed in the figure legend below). The differences between the values were tested by repeated one-way ANOVA measurements. The correlation between the values was tested using Spearman’s test.

For each ROI (chest, abdomen, and forehead) a "thermal" value and an "entropy" value were measured in each image throughout the effort. Comparisons were made between the different body regions (temperature and entropy) and the changes obtained in VE, AT and RC points.

All statistical analyses were performed using GraphPad Prism version 8.00 (GraphPad Software, La Jolla, CA, USA) and MATLAB software (Mathworks Inc. Natick, MA, USA).

## Results

**Figure 2:**
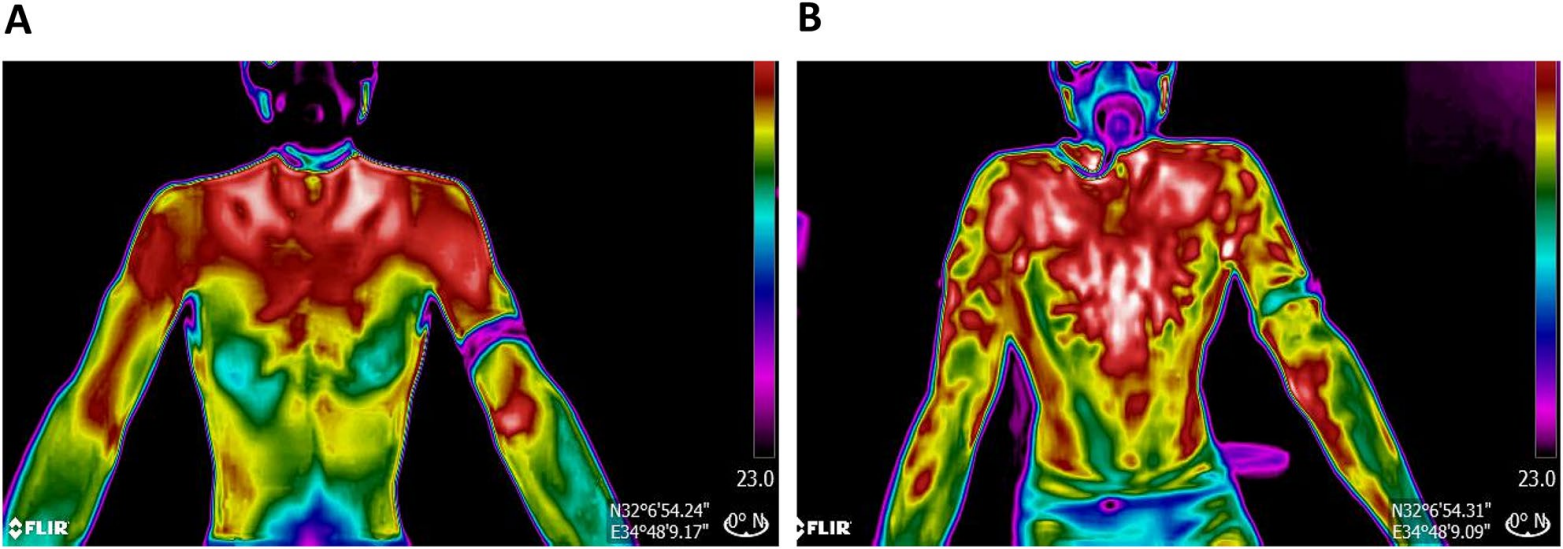
A thermal image of a representative participant (**A**) at the beginning of the exercise test and (**B**) at the end of the exercise test where the tree-shaped hotspot areas can be seen.

Eighteen subjects performed the exercise stress test for a mean exercise duration of 10.04 ± 0.1 min. The maximum heart rate was 189.7 ± 8.7 bpm, and VO2 max (maximal oxygen consumption) 41.5 ± 6.7 ml/kg/min. Thermal images were captured throughout the exercise test (Fig. 2).

**Figure 3:**
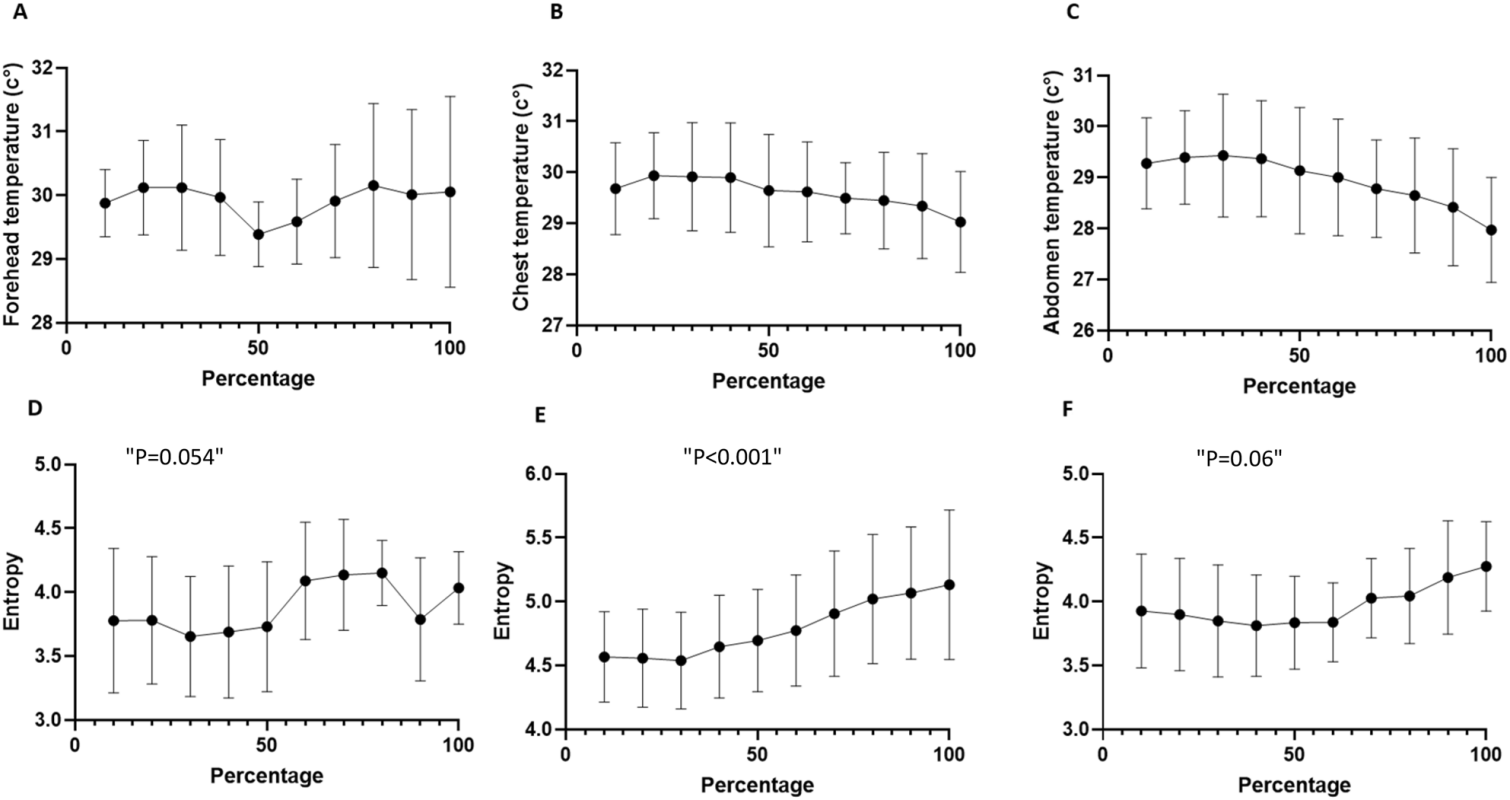
Mean temperature and entropy of the ROIs during the exercise test. The x-axis represents the percentage of the exercise test time. The y- axis represents (**A**) the forehead temperature, (**B**) the chest temperature, (**C**) the abdomen temperature, (**D**) the forehead entropy, (**E**) the chest entropy, and (**F**) the abdomen entropy.

Figure 3 shows the mean skin temperature and entropy.

Skin temperatures neither changed during the exercise test, nor in the head, chest, or abdomen regions. However, the entropy of the chest surface area increased significantly throughout the exercise test compared with baseline temperature at rest. At the beginning of the exercise, the mean entropy was 4.57 ± 0.35 and reached a maximum of 5.13 ± 0.58 at peak exercise. This increase in entropy was correlated with exercise duration and intensity (Spearman’s test: r = 0.9515, p < 0.001). The abdominal entropy started at 3.93 ± 0.45 at rest and increased to a maximum value of 4.27 ± 0.35 (Spearman’s test: r = 0.62, p = 0.06). The entropy of the forehead surface was 3.78 ± 0.56 at rest and 4.03 ± 0.28 at peak exercise (Spearman’s test : r = 0.63, p = 0.054).

By using one-way ANOVA, the increase in entropy was found to be statistically significant for the chest (p < 0.001) and abdomen (p = 0.006) but not for the forehead (p = 0.17).

**Figure 4:**
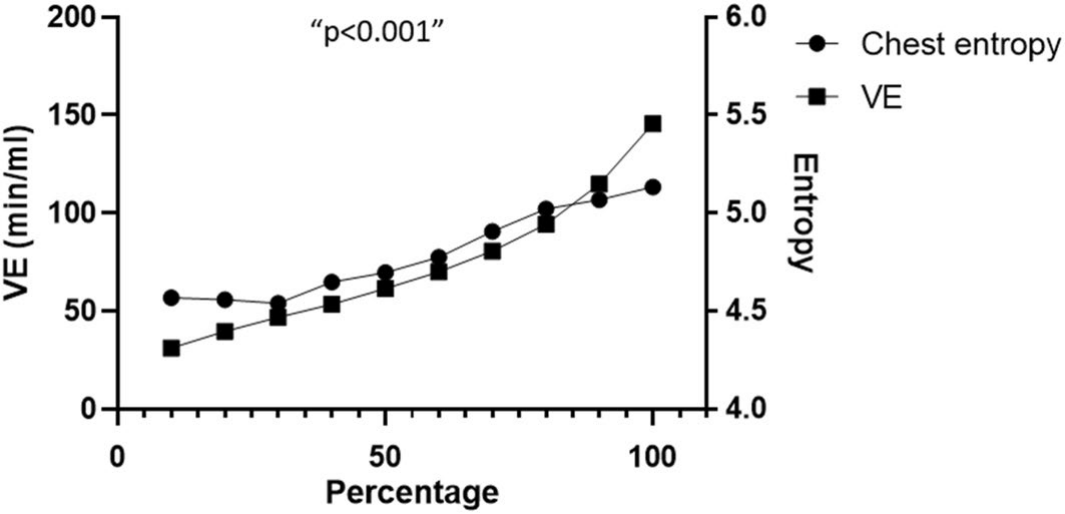
VE and entropy increases during the exercise test. The x-axis represents the percentage of the exercise test time.

Figure 4 shows the correlation between the increase in VE and the increase in the chest entropy during exercise. This correlation was statistically significant (r = 0.9515, p < 0.001). No correlations were found between the increase in entropy and the abdomen or the forehead compared with the VE.

**Figure 5:**
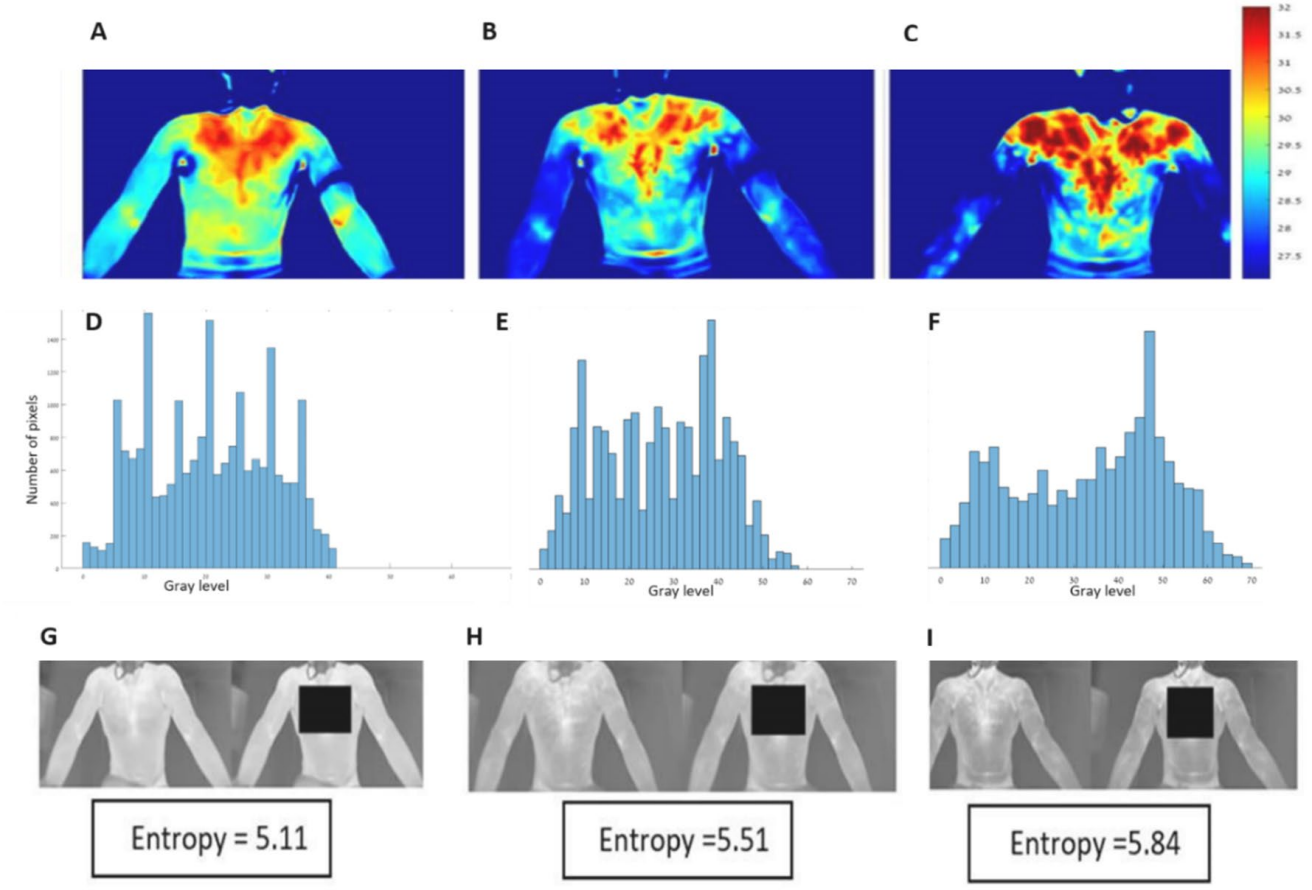
The thermal images and entropy as observed at three time points in the exercise test. (**A**, **D**, **G**) at the beginning of the exercise (before the aerobic threshold), (**B**, **E**, **H**) between the aerobic threshold (AT) and the respiratory compensation point (RCP), and (**C**, **F**, **I**) towards the end of the exercise (after the RCP). The bottom row of figures shows the manually marked ROI.

The histogram shows the gray levels in the image. It can be seen that throughout the effort, the division between the gray levels in the image (representing temperature distribution), as well as the entropy, increased (Fig. 5).

No correlations were found between body temperature and entropy, for all three body regions, with AT or RC points.

## Discussion

In our preliminary study, we examined the temperature changes and the temperature distribution (entropy) of the torso during graded cycling exercise stress test using thermal imaging. We found a significant increase in entropy that was correlated with exercise intensity. Furthermore, we found a high correlation between the changes in chest entropy and a proportional increase in pulmonary ventilation (VE).

Current reports using infrared thermography during exercise have primarily focused on measuring absolute body temperature. However, these attempts have demonstrated limited effectiveness, and almost no significant correlations have been found with physiological parameters^1,14^. In the current study, we found no statistical changes in surface temperatures during the incremental exercise test. The considerable differences in surface temperatures during incremental exercise test can be related to changes in the ROI determination, measurement methods, and the differences in exercise test protocols^1,7,15,16^. Additionally, the temperature is sensitive to environmental changes, such as ambient temperature and humidity, sweating degree, and body hair amount in the ROI.

Our innovative approach of extracting advanced texture and shape features from thermal images enhances the impact of our study findings. In a recent study by Brzezinski et al., SARS-CoV-2 infection was diagnosed using thermal imaging. In this case, no significant thermal changes were found in absolute temperature values. However, a significant difference was observed in the calculated textural features (entropy), contrast, and homogeneity^17^. Another unrelated study also demonstrated the same concept in an animal model of fatty liver^18^.

The fact that mean surface temperature at the ROI did not correlate with the increase in the exercise and muscle work in the test group supports our rationale to focus on temperature distribution across organs of interest instead of a standard absolute temperature assessment. Entropy is a statistical measure that expresses the texture of an image and is less sensitive to environmental changes than to absolute temperature.

These entropy changes, manifested as a difference in the image's texture, were visually described in previous studies. Studies on this subject described the warmer areas formed, also known as "tree shapes, hyperthermal zones, or even vessel shapes", during the exercise test^10–12^ (Fig. 3). In thermal photography, these areas appeared brighter (warmer) and created a characteristic image reminiscent of a tree and its branches or a superficial vascular system. It has been claimed that the tree-shaped pattern represents perforator vessels^1,12^, and its formation is due to the opening of superficial blood vessels during exercise. There is an increase in the internal and exercising tissue temperature during exercise, which causes reflex neurogenic vasodilation. This leads to the active opening and vasodilation of the vessels in the superficial vascular system of the skin^19–21^. This study quantitatively describes the changes in the texture obtained through thermal imaging, and significant changes in the entropy measurement were found throughout the exercise.

Furthermore, we found that the increase in chest entropy was highly correlated with the increase in pulmonary ventilation during exercise. During an incremental aerobic exercise stress test, respiratory muscles in the chest are active and increase their oxygen consumption. The increase in oxygen consumption of the chest muscles in exertion can reach a rate of 10–15% of the total oxygen consumption in the body^22^. This increase is accompanied by changes in the heat produced by and emitted from the active muscles^23^. The increase in heat production in the active muscles correlates with the biogenesis of ATP production, which can double during high-intensity exercise^23^. Increased metabolic heat production leads to vasodilatation and the opening of superficial blood vessels in these areas. This is supported by a previous study that demonstrated that active muscle areas presented a greater blood flow and had higher temperatures than other areas, such as the chest area being warmer than the back^10^. Therefore, it can be assumed that the increased work of breathing by the chest muscles during exertion, which is expressed as an increase in pulmonary ventilation, led to the opening of superficial blood vessels, changing the entropy in the chest.

In conclusion, surface body temperature measured during incremental exercise stress test using IR camera showed no significant changes in skin temperature of the limbs, chest, and forehead. However, we found a significant increase in the entropy measurement throughout the exercise, which was correlated with pulmonary ventilation. The entropy findings are consistent with other studies that have described the “hot spotted areas” phenomenon. The present study is the first to describe this phenomenon quantitatively. Further studies are required to investigate whether this approach can detect ventilatory anaerobic thresholds remotely using an IR camera.

However, this study has several limitations, namely, (1) only male participants were involved in testing (because bare chest images needed to be acquired); (2) all the study participants belonged to the same age range (20 to 30 years); (3) FLIR ONE resolution limitation can interfere with results, and future research with a higher quality camera may lead to better results. (4) all the study participants were healthy and physically active. Further research with a larger sample, including more diverse populations, will be needed to apply this measurement method clinically.
